# Depressive Symptoms, Sleep Quality and Diet During the 2019 Novel Coronavirus Epidemic in China: A Survey of Medical Students

**DOI:** 10.3389/fpubh.2020.588578

**Published:** 2021-01-26

**Authors:** Jianping Xie, Xia Li, Haiyun Luo, Liu He, Yufan Bai, Fuyun Zheng, Lanchun Zhang, Jiaqing Ma, Zhiqiang Niu, Yubing Qin, Ling Wang, Wenjie Ma, Haofei Yu, Rongping Zhang, Ying Guo

**Affiliations:** ^1^School of Basic Medical Sciences, Kunming Medical University, Kunming, China; ^2^School of Pharmaceutical Science, Department of Zoology & Yunnan Key Laboratory of Pharmacology for Natural Products, Kunming Medical University, Kunming, China; ^3^Department of Technology, Library, Yunnan Minzu University, Kunming, China; ^4^School of Chinese Materia Medica and Yunnan Key Laboratory of Southern Medicinal Resources, Yunnan University of Chinese Medicine, Kunming, China

**Keywords:** academic stress, employment pressure, COVID-19, medical students, depression

## Abstract

The psychological condition of medical students may be influenced by the 2019 novel coronavirus (COVID-19) outbreak. This study investigated the prevalence and influencing factors of depressive symptoms, poor sleep quality and poor diet in students at Kunming Medical University during the early part of the COVID-19 outbreak. A cross-sectional study was used from a questionnaire survey in February 2020. Of a total of 1,026 study participants, the prevalence of depressive symptoms, poor sleep quality, and poor diet was, respectively, 22.4, 33.2, and 17.4%. Male students and students with a low degree of focus on COVID-19 had a high risk of depressive symptoms. A high percentage of females and students in the fifth grade, as well as students with high levels of concern about the negative impact of COVID-19 on their education or employment, comprised those with poor sleep quality. Students in the fifth grade and students with high levels of concern about the negative impact of COVID-19 on their education or employment were more likely to report poor diet. This study suggests the importance of monitoring medical students' depressive state during the COVID-19 outbreak, and universities are encouraged to institute policies and programs to provide educational counseling and psychological support to help students to cope with these problems.

## Introduction

According to epidemiological data published in Nature, the prevalence of depression in China was reported to be 3.02% (1). The lifetime prevalence of depressive disorders was 6.9%, while the 12-months prevalence of depressive disorders was 3.6% (2). The prevalence of depressive disorder was 8.8% of the study population among college students in China (3). The prevalence of depression among medical students in China has been reported with a mean of 32.74%, based on a published meta-analysis (4). In America, the prevalence rates of depression in college students ranged from 7 to 9% (5), while the prevalence of 6.0–66.5% for depression in medical students was recorded (6). Additionally, the number of students at universities who suffered from mental problems was increasing (7). Since December 2019, cases of pneumonia have appeared in Wuhan city, Hubei Province, China (8, 9). This kind of pneumonia is a highly contagious disease caused by the 2019 novel coronavirus (COVID-19), which is characterized by a rapid transmission. Among those who developed symptoms, about 15% became seriously ill and 5% became critically ill. Complications leading to death may include respiratory failure, acute respiratory distress syndrome, sepsis and septic shock, thromboembolism, and multiorgan failure^1^. The impact of COVID-19 in China was even greater than that of severe acute respiratory syndrome (SARS) in 2002. One study suggested that the SARS outbreak showed a psychosocial influence on Chinese students, even though none of them became infected SARS (10). Depression accompanied major sleep problems in post-SARS syndrome was reported (11). Insomnia was both a frequent symptom and a risk factor of depression (12). The positive relationships between a well-diet and sleep state and improved human health was also documented (13). According to this evidence, we presume that the psychological condition, the sleep quality and the diet state of medical students may be influenced by the COVID-19 outbreak.

Although there have been many empirical studies about depression in medical students in China, few studies to date have conducted a survey on the depressive symptoms, the sleep quality and the diet state of medical students at Kunming Medical University during the COVID-19 pneumonia outbreak. Therefore, the purpose of this study is to perform a cross-sectional study of the prevalence and influencing factors of depressive symptoms, sleep quality and diet during the early COVID-19 outbreak and to lay the groundwork for further measures for at-risk medical students.

## Methods

### Study Design and Participants

The study was designed to investigate the subjects' depressive symptoms, sleep quality and diet. To select a representative sample of respondents, we distributed an invitation to clinical medical undergraduates to complete the web-based questionnaire. This web-based questionnaire was sent to the WeChat public platform. All invited students may see this questionnaire and may answer it by scanning the quick response code. This web-based survey was non-commercial, anonymous, and completely voluntary. This was a cross-sectional design. We adopted a stratified sampling method to recruit the clinical medical undergraduates of the University from students in the first, second, third, fourth and fifth grade. Non-clinical majors within the same university such as nursing and medical imaging were excluded. Students who met with the criteria were all invited to complete the questionnaire. The estimated sample sizes required for the first, second, third, fourth and fifth grade were respectively 167, 160, 164, 163, and 161. In total, 1026 respondents completed the questionnaire. The final sample composed of 244 (23.8%) first grade students, 182 (17.7%) second grade students, 225 (21.9%) third grade students, 229 (22.3%) fourth grade students, and 146 (14.2%) fifth grade students.

### Data Collection

This study was conducted during the early outbreak of COVID-19 in February 2020, and data were collected from February 18 to February 22 through online questionnaires. The questionnaire included three parts: basic information, COVID-19-related information, and assessment of depressive state, sleep quality and diet. The final study population consisted of a total of 1,026 undergraduate students majoring in clinical medicine.

### Measurements

#### Basic Information

Demographic variables included gender (male or female), grade group (first grade, second grade, third grade, fourth grade, or fifth grade), age group (16–18, 19–21, 22–24, or 25–26 years), and location (five named provinces or other). Because more clinical medicine students at Kunming Medical University were females than males, more females than males were enrolled in the study. Because more students were from Yunnan Province, the number of students from five provinces, including Hubei, Guangdong, Henan, Zhejiang and Hunan provinces, was significantly lower than the number of students from other provinces in China. The selection of these five provinces was based on the cumulative confirmed number of novel coronavirus pneumonia cases >1,000 on February 22, 2020.

#### Depressive Symptoms

The modified Chinese version of the Self-Rated Depression scale was used to evaluate the outcomes of depressive symptoms over the past 2 weeks. The scale was composed of 20 multiple choice questions. The total score ranged from 0 to 60, and a total score of >22 was defined as indicative of depressive symptoms.

#### Sleep Quality

We used the modified Chinese version of the Pittsburgh Sleep Quality Index scale to measure students' sleep quality over the past 2 weeks. The total score ranged from 0 to 21, and a score >7 was defined as indicative of poor sleep quality.

#### State of Diet

The modified Chinese version of the state of diet scale was applied to assess the students' diet. The scale contained items on loss of appetite, overeating, constipation, etc. The total score ranged from 0 to 15, and a score >5 was defined as indicative of poor diet.

#### Degree of Focusing on the COVID-19 or Concerned About Negative Impact of COVID-19 on Education or Employment

A series of questions related to emotions were applied to assess the degree of focusing on the COVID-19 or concerned about negative impact of COVID-19 on education or employment. The total score ranged from 0 to 20, and a score >6 was defined as indicative of high degree of focusing on the COVID-19 or concerned about negative impact of COVID-19 on education or employment.

### Statistical Analysis

The data were analyzed using chi-square tests (χ^2^) to compare the differences between groups and univariate and multivariate logistic regression models to seek potential influencing factors for depressive symptoms, sleep quality and diet during the COVID-19 outbreak. *P*-values were two-sided and considered significant at *P* < 0.05. All analyses were conducted using SPSS 19.0 (SPSS Inc., Chicago, IL, USA).

## Results

### Demographic Characteristics

Table 1 presents the general characteristics of the study population. Of a total of 1,026 study participants, 373 (36.4%) were males and 653 (63.6%) were females. Among these participants, 244 (23.8%) students were in the first grade, 519 (50.6%) students were 19–21 years of age, and 1,007 (98.1%) students were located in provinces other than the five provinces of interest (Hubei, Guangdong, Henan, Zhejiang, and Hunan provinces). A total of 710 (69.2%) students were highly focused on COVID-19. A total of 608 (59.3%) students were highly concerned about the negative impact of COVID-19 on their education or employment.

**Table 1:** Demographic characteristics of study sample (*N* = 1,026).

**Variable**	***n* (%)**
**GENDER**	
Male	373 (36.4%)
Female	653 (63.6%)
**GRADE**	
First	244 (23.8%)
Second	182 (17.7%)
Third	225 (21.9%)
Fourth	229 (22.3%)
Fifth	146 (14.2%)
**AGE**	
16–18	87 (8.5%)
19–21	519 (50.6%)
22–24	406 (39.6%)
25–26	14 (1.4%)
**LOCATION**	
Five provinces	19 (1.9%)
Other provinces	1,007 (98.1%)
**DEGREE OF FOCUSING ON THE COVID-19**	
High	710 (69.2%)
Low	316 (30.8%)
**DEGREE OF CONCERNED ABOUT NEGATIVE IMPACT OF COVID-19 ON EDUCATION OR EMPLOYMENT**	
High	608 (59.3%)
Low	418 (40.7%)
**Total**	1,026 (100%)

### Prevalence of Depressive Symptoms, Sleep Quality, and Diet During the COVID-19 Outbreak in Medical Students, Stratified by Gender, Grade, Age, and Location

Tables 2–5 presents the prevalence of depressive symptoms, poor sleep quality, and poor diet during the COVID-19 outbreak in medical students, who were stratified by gender, grade, age, and location. The prevalence of depressive symptoms was significantly higher in males than in females (*P* < 0.05, Table 2), while the prevalence of poor sleep quality was significantly higher in females than in males (*P* < 0.05, Table 2). Compared with students in other grades, students in the fifth grade reported the highest rate of poor sleep quality and poor diet (*P* < 0.05, Table 3). There was no significant difference in the prevalence of depressive symptoms, poor sleep quality, or poor diet by age (*P* > 0.05, Table 4) or by location (*P* > 0.05, Table 5).

**Table 2:** Prevalence of depressive symptoms, sleep quality, and state of diet during COVID-19 outbreak in medical students stratified by gender (*N* = 1,026).

**Variables**	**Male (*N =* 373)**	**Female (*N =* 653)**	**Total (*N =* 1,026)**	**χ^**2**^**	***P*-value**
	***n* (%)**	***n* (%)**	***n* (%)**		
Depressive symptoms				5.73	0.017
No	274 (73.5%)	522 (79.9%)	796 (77.6%)		
Yes	99 (26.5%)	131 (20.1%)	230 (22.4%)		
Sleep quality				5.47	0.019
Good	266 (71.3%)	419 (64.2%)	685 (66.8%)		
Poor	107 (28.7%)	234 (35.8%)	341 (33.2%)		
State of diet				2.41	0.121
Good	317 (85.0%)	530 (81.2%)	847 (82.6%)		
Poor	56 (15.0%)	123 (18.8%)	179 (17.4%)		

**Table 3:** Prevalence of depressive symptoms, sleep quality, and state of diet during COVID-19 outbreak in medical students stratified by grade (*N* = 1,026).

**Variables**	**First (*N =* 244)**	**Second (*N =* 182)**	**Third (*N =* 225)**	**Fourth (*N =* 229)**	**Fifth (*N =* 146)**	**Total (*N =* 1026)**	**χ^**2**^**	***P*-value**
	***n* (%)**	***n* (%)**	***n* (%)**	***n* (%)**	***n* (%)**	***n* (%)**		
Depressive symptoms							4.32	0.364
No	194 (79.5%)	140 (76.9%)	171 (76.0%)	185 (80.8%)	106 (72.6%)	796 (77.6%)		
Yes	50 (20.5%)	42 (23.1%)	54 (24.0%)	44 (19.2%)	40 (27.4%)	230 (22.4%)		
Sleep quality							21.27	<0.001
Good	189 (77.5%)	116 (63.7%)	145 (64.4%)	153 (66.8%)	82 (56.2%)	685 (66.8%)		
Poor	55 (22.5%)	66 (36.3%)	80 (35.6%)	76 (33.2%)	64 (43.8%)	341 (33.2%)		
State of diet							20.23	<0.001
Good	216 (88.5%)	144 (79.1%)	183 (81.3%)	198 (86.5%)	106 (72.6%)	847 (82.6%)		
Poor	28 (11.5%)	38 (20.9%)	42 (18.7%)	31 (13.5%)	40 (27.4%)	179 (17.4%)		

**Table 4:** Prevalence of depressive symptoms, sleep quality, and state of diet during COVID-19 outbreak in medical students stratified by age (*N* = 1,026).

**Variables**	**16–18 (*N =* 87)**	**19–21 (*N =* 519)**	**22–24 (*N =* 406)**	**25–26 (*N =* 14)**	**Total (*N =* 1,026)**	**χ^**2**^**	***P*-value**
	***n* (%)**	***n* (%)**	***n* (%)**	***n* (%)**	***n* (%)**		
Depressive symptoms						0.74	0.864
No	70 (80.5%)	402 (77.5%)	314 (77.3%)	10 (71.4%)	796 (77.6%)		
Yes	17 (19.5%)	117 (22.5%)	92 (22.7%)	4 (28.6%)	230 (22.4%)		
Sleep quality						6.67	0.083
Good	62 (71.3%)	360 (69.4%)	256 (63.1%)	7 (50.0%)	685 (66.8%)		
Poor	25 (28.7%)	159 (30.6%)	150 (36.9%)	7 (50.0%)	341 (33.2%)		
State of diet						3.52	0.319
Good	77 (88.5%)	428 (82.5%)	332 (81.8%)	10 (71.4%)	847 (82.6%)		
Poor	10 (11.5%)	91 (17.5%)	74 (18.2%)	4 (28.6%)	179 (17.4%)		

**Table 5:** Prevalence of depressive symptoms, sleep quality, and state of diet during COVID-19 outbreak in medical students stratified by location (*N* = 1,026).

**Variables**	**Other provinces (*N =* 1,007)**	**Five provinces (*N =* 19)**	**Total (*N =* 1,026)**	**χ^**2**^**	***P*-value**
	***n* (%)**	***n* (%)**	***n* (%)**		
Depressive symptoms				0.02	0.886
No	781 (77.6%)	15 (78.9%)	796 (77.6%)		
Yes	226 (22.4%)	4 (21.1%)	230 (22.4%)		
Sleep quality				0.69	0.407
Good	674 (66.9%)	11 (57.9%)	685 (66.8%)		
Poor	333 (33.1%)	8 (42.1%)	341 (33.2%)		
State of diet				2	0.158
Good	829 (82.3%)	18 (94.7%)	847 (82.6%)		
Poor	178 (17.7%)	1 (5.3%)	179 (17.4%)		

### Association of Influencing Factors With Depressive Symptoms, Poor Sleep Quality, and Poor Diet During the COVID-19 Outbreak

Table 6 presents the association of influencing factors of depressive symptoms, poor sleep quality, and poor diet during the COVID-19 outbreak. In the univariate logistic regression models, gender (OR = 1.45, 95% CI: 1.07–1.96) and degree of focus on COVID-19 (OR = 1.47, 95% CI: 1.08–2.00) were significantly associated with depressive symptoms. Gender (OR = 0.72, 95% CI: 0.55–0.95), grade (OR = 2.68, 95% CI: 1.72–4.18) and degree of concern about the negative impact of COVID-19 on education or employment (OR = 2.10, 95% CI: 1.59–2.77) were related to poor sleep quality during the COVID-19 outbreak in medical students. Similarly, grade (OR = 2.91, 95% CI: 1.70–4.98) and degree of concern about the negative impact of COVID-19 on education or employment (OR = 1.60, 95% CI: 1.13–2.25) were associated with poor diet, but gender was not associated with poor diet (OR = 0.76, 95% CI: 0.54–1.08).

**Table 6:** Results of univariate and multivariate logistic regression analyses (*N* = 1,026).

**Variable**	**Depressive symptoms**		**Sleep quality**		**State of diet**	
	**OR (95% CI)**	**AOR (95% CI)**	**OR (95% CI)**	**AOR (95% CI)**	**OR (95% CI)**	**AOR (95% CI)**
**GENDER**						
Male	1.45 (1.07, 1.96)^*^	1.42 (1.05, 1.93)^*^	0.72 (0.55, 0.95)^*^	0.74 (0.56, 0.98)^*^	0.76 (0.54, 1.08)	0.76 (0.53, 1.08)
Female	1.00	1.00	1.00	1.00	1.00	1.00
**GRADE**						
First	1.00	1.00	1.00	1.00	1.00	1.00
Second	1.16 (0.73, 1.85)	1.18 (0.71, 1.96)	1.96 (1.28, 2.99)^*^	2.36 (1.45, 3.87)^*^	2.04 (1.20, 3.47)^*^	2.05 (1.14, 3.72)^*^
Third	1.23 (0.79, 1.90)	1.27 (0.76, 2.13)	1.90 (1.26, 2.85)^*^	2.21 (1.34, 3.64)^*^	1.77 (1.06, 2.97)^*^	1.85 (1.01, 3.40)^*^
Fourth	0.92 (0.59, 1.45)	0.90 (0.49, 1.66)	1.71 (1.14, 2.57)^*^	1.56 (0.89, 2.73)	1.21 (0.70, 2.09)	1.21 (0.59, 2.45)
Fifth	1.46 (0.91, 2.36)	1.45 (0.72, 2.91)	2.68 (1.72, 4.18)^*^	2.26 (1.19, 4.29)^*^	2.91 (1.70, 4.98)^*^	2.92 (1.33, 6.39)^*^
**AGE**						
16–18	0.84 (0.21, 3.47)	0.74 (0.18, 3.06)	0.40 (0.13, 1.27)	1.01 (0.28, 3.67)	0.33 (0.09, 1.23)	0.81 (0.18, 3.70)
19–21	0.91 (0.26, 3.23)	0.82 (0.23, 2.90)	0.44 (0.15, 1.28)	0.60 (0.19, 1.90)	0.53 (0.16, 1.73)	0.85 (0.23, 3.08)
22–24	0.85 (0.26, 2.84)	0.77 (0.23, 2.56)	0.59 (0.20, 1.70)	0.61 (0.20, 1.83)	0.56 (0.17, 1.83)	0.65 (0.19, 2.20)
25–26	1.00	1.00	1.00	1.00	1.00	1.00
**LOCATION**						
Other provinces	1.02 (0.33, 3.11)	0.96 (0.31, 2.98)	0.68 (0.27, 1.71)	0.73 (0.28, 1.90)	3.87 (0.51, 29.14)	3.68 (0.48, 28.13)
Five provinces	1.00	1.00	1.00	1.00	1.00	1.00
**DEGREE OF FOCUSING ON THE COVID-19**						
Low	1.47 (1.08, 2.00)^*^	1.47 (1.07, 2.02)^*^	1.00 (0.75, 1.32)	0.90 (0.67, 1.21)	1.13 (0.80, 1.59)	1.09 (0.76, 1.57)
High	1.00	1.00	1.00	1.00	1.00	1.00
**DEGREE OF CONCERNED ABOUT NEGATIVE IMPACT OF COVID-19 ON EDUCATION OR EMPLOYMENT**						
Low	1.00	1.00	1.00	1.00	1.00	1.00
High	1.12 (0.83, 1.52)	1.05 (0.75, 1.47)	2.10 (1.59, 2.77)^*^	2.12 (1.55, 2.88)^*^	1.60 (1.13, 2.25)^*^	1.55 (1.06, 2.28)^*^

* *P < 0.05*.

The associations weakened, but there were still significant differences in the multivariate logistic regression models. Male students (AOR = 1.42, 95% CI: 1.05–1.93) and students focusing less on COVID-19 (AOR = 1.47, 95% CI: 1.07–2.02) had a higher risk of depressive symptoms than did female students and students highly focused on COVID-19, respectively. Compared with females, males were less likely to have poor sleep quality (AOR = 0.74, 95% CI: 0.56–0.98). Students in the fifth grade (AOR = 2.26, 95% CI: 1.19–4.29) and students with high concerns about the negative impact of COVID-19 on their education or employment (AOR = 2.12, 95% CI: 1.55−2.88) were more likely to have poor sleep quality than were students in other grades and students without concerns about the negative impact of COVID-19 on education or employment, respectively. In addition, compared with students in other grades and students without concerns about the negative impact of COVID-19 on education or employment, students in the fifth grade (AOR = 2.92, 95% CI: 1.33–6.39) and students with high concerns about the negative impact of COVID-19 on education or employment (AOR = 1.55, 95% CI: 1.06–2.28) were more likely to report poor diet.

## Discussion

Our study showed that the prevalence of depressive symptoms, poor sleep quality, and poor diet was 22.4, 33.2, and 17.4%, respectively. Male students and students with low COVID-19 scores had a higher risk of depressive symptoms. Females, students in the fifth grade, and students with high concerns about the negative impact of COVID-19 on their education or employment were more likely to have poor sleep quality. Students in the fifth grade and students with high concerns about the negative impact of COVID-19 on their education or employment were more likely to report poor diet.

The questionnaire survey showed that over one-fifth of the undergraduates had depressive symptoms. This result is in accordance with the range reported in a previous study for Chinese medical students (4). Our survey revealed that the incidence of depressive symptoms in male students was higher than that in female students, although previous research has shown that depression was more prevalent in female students than in male students (14). One possible reason for this finding may be males are not good at releasing pressure. It is suggested that males take part in outdoor activities appropriately, do sports regularly, relax their requirements on themselves and establish beneficial interests and hobbies, which can release pressure and regulate physical and mental health.

Studies have reported that an increased risk of depressive symptoms was associated with poor sleep quality (15). A study from China found that 34.3% of middle-school students had sleep problems (16). Our findings showed that over one-third of female students had poor sleep, which was significantly higher than the corresponding estimate for male students. One possible reason for this finding may be the differences between men and women in facing stress (17). Females achieved at significantly higher level than males, in regard to detaching themselves from the emotions of a situation (18). Additionally, coping strategies of women included more frequent social and emotional support (19). For females, the ruminative coping style was generally adopted, whereas males used the distractive coping style (20). Female gender showed the worst condition for sleep quality and insomnia during the home confinement period in Italian (21). Therefore, greater chronic stress and psychological distress during the COVID-19 outbreak may cause more sleep problems for female students than for male students. Healthy sleep lectures are held for female students, and it is suggested that they arrange their study plan and rest time reasonably, and carry out psychological counseling timely.

We found that grade was a potential risk factor for poor sleep quality and poor diet, and fifth graders had the highest incidence of poor sleep and poor diet. A study reported that senior medical students grapple with fatigue caused by unprecedented levels of sleep deprivation (22). One reason for poor sleep and poor diet in fifth-grade students may be greater employment or examination pressures they are facing. In turn, poor sleep quality has a negative impact on academic performance (23), which aggravates academic stress. Medical students with large employment pressure showed more anxiety symptoms (24). Additionally, a study in Mexico reported that depressed medical students showed a combination of severe academic stress and sleep problems (25). Psychosocial stress and abnormal diet (loss of appetite or overeating) are interrelated epidemiological phenomena that are already present in youth (26). Another study suggested that the neural mechanism of acute stress affecting appetite was by the frontal pole (27). Direct or indirect stress from COVID-19 may be the cause of abnormal diet during the COVID-19 outbreak.

Meanwhile, the degree of concern about the negative impact of COVID-19 on education or employment was a potential risk factor for poor sleep quality and poor diet. Students who felt more academic stress or employment pressure during the COVID-19 outbreak had poor sleep and a poor diet. It is possible that the severe impact of the novel coronavirus pneumonia outbreak aggravates the academic stress or employment pressure faced by the students. COVID-19 affects the lower respiratory tract and manifests as pneumonia in humans. The breath exhaled by patients with pneumonia carries many viruses, and its droplets are very infectious (28). The continuous increase in the incidence of novel coronavirus pneumonia has seriously affected the everyday lives of Chinese people, although the incidence in Yunnan Province is low. The increasing academic or employment stress may also indicate that to stop the spread of COVID-19, the government must take measures of closure, which will delay the students' return to school and make it difficult for graduates to find jobs. For the fifth grade students who are looking for jobs, the university will hold online job fairs to provide more research assistant positions, track the employment situation of graduates, and provide recruitment information to graduates in time to alleviate the negative impact of COVID-19 on employment. For fifth-year students who are preparing to take the postgraduate entrance examination, the state has eased the pressure of admission by expanding the enrollment scale and providing online entrance examination.

Fortunately, during the epidemic outbreak at our university in February 2020, all undergraduate students were spending their winter vacation at home. The vacation exerted a positive impact on population health with regard to depressive symptoms (29, 30). Additionally, medical students have a thorough knowledge of the regulation of medicines and the development of diseases, and therefore, they mastered ways to cope with the epidemic situation. After encountering the epidemic, non-medical students possibly think more about how to reduce the impact of the epidemic on themselves and their families. More medical students think about how to use what they have learned to help others understand COVID-19, how to guide others to take reasonable precautions to avoid transmission and infection of COVID-19, and how to help people relieve anxiety and tension during the epidemic. There has been no large-scale outbreak in Yunnan and the medical system is functioning normally. After the outbreak, medical students received more detailed training in epidemiology and clinical skills. Compared to medical students in Vietnam (31), medical students at Kunming Medical University provide support for the covid-19 response by means of science popularization, auxiliary psychological counseling and epidemiological data collection. Moreover, in response to the outbreak of the epidemic, the university immediately suspended classes and used online courses to replace traditional classroom teaching. For graduating students, the use of online tools to find employment instead of finding a job face-to-face has solved the practical difficulties of graduates. To relieve the various aspects of pressure faced by medical students, it is suggested that the university build a network psychological counseling room to help students cope with mental health challenges.

This study has two limitations. First, the duration of this study was short. However, the objective of this study was to assess the immediate depressive symptoms, poor sleep quality, and poor diet during the early COVID-19 outbreak. In subsequent studies, we will further track the depressive state of medical students over a longer period of time. Second, the subjects in this study were medical students at Kunming Medical University, which is restricted by the region. The research area should be expanded to include medical students in all medical universities in China. However, the findings in this study still suggest the importance of monitoring medical students' mental health during the novel coronavirus epidemic. Measures should be strengthened to reduce the stress of novel coronavirus pneumonia and other factors in medical students.

## Conclusions

In conclusion, our study identified a major mental health burden of medical students during the COVID-19 outbreak. Male students and students with a low degree of focus on COVID-19 had a high risk of depressive symptoms. A high percentage of females and students in the fifth grade, as well as students with high levels of concern about the negative impact of COVID-19 on their education or employment, comprised those with poor sleep quality. Students in the fifth grade and students with high levels of concern about the negative impact of COVID-19 on their education or employment were more likely to report a poor diet. This study suggests the importance of monitoring medical students' depressive state during the early part of the COVID-19 outbreak, and universities are encouraged to institute policies and programs to provide educational counseling and psychological support to help students cope with these problems.

## Data Availability Statement

The raw data supporting the conclusions of this article will be made available by the authors, without undue reservation.

## Ethics Statement

The study was approved by the Research Ethics Committees of Kunming Medical University, Yunnan Province, China, and conducted in accordance with the Declaration of Helsinki principles. Informed consent from all participants was included at the start of the questionnaire, and all participants voluntarily participated or withdrew from the questionnaire.

## Author Contributions

JX, XL, HL, RZ, and YG: Conceptualization and formal analysis. YB, LH, FZ, LZ, and JM: Data curation. HL, HY, RZ, and YG: Funding acquisition. ZN, YQ, LW, WM, and HY: Methodology. JX, XL, and HL: Writing—original draft. RZ and YG: Writing—review & editing. All authors contributed to the article and approved the submitted version.

## Conflict of Interest

The authors declare that the research was conducted in the absence of any commercial or financial relationships that could be construed as a potential conflict of interest.

## Footnotes

^1^www.who.int/emergencies/diseases/novel-coronavirus-2019

## References

[1] Smith K. Mental health: a world of depression. Nature. (2014) 515:181. 10.1038/515180a 25391942

[2] Huang Y Wang Y Wang H Liu Z Yu X Yan J Prevalence of mental disorders in china: a cross-sectional epidemiological study. Lancet Psychiatry. (2019) 6:211–24. 10.1016/s2215-0366(18)30511-x 30792114

[3] Zhang YL Liang W Chen ZM Zhang HM Zhang JH Weng XQ. Validity and reliability of patient health questionnaire-9 and patient health questionnaire-2 to screen for depression among college students in China. Asia Pac Psychiatry. (2013) 5:268–75. 10.1111/appy.12103 24123859

[4] Mao Y Zhang N Liu J Zhu B He R Wang X. A systematic review of depression and anxiety in medical students in China. BMC Med Educ. (2019) 19:327. 10.1186/s12909-019-1744-2 31477124 PMC6721355

[5] Eisenberg D Hunt J Speer N. Mental health in american colleges and universities: variation across student subgroups and across campuses. J Nerv Ment Dis. (2013) 201:60–7. 10.1097/NMD.0b013e31827ab077 23274298

[6] Hope V Henderson M. Medical student depression, anxiety and distress outside north america: a systematic review. Med Educ. (2014) 48:963–79. 10.1111/medu.12512 25200017

[7] Pedrelli P Nyer M Yeung A Zulauf C Wilens T. College students: mental health problems and treatment considerations. Acad Psychiatry. (2015) 39:503–11. 10.1007/s40596-014-0205-9 25142250 PMC4527955

[8] Rubin EJ Baden L R Morrissey S Campion EW Medical journals and the 2019-ncov outbreak. N Engl J Med. (2020) 382:866 10.1056/NEJMe2001329 31986242

[9] Lancet. The emerging understandings of 2019-Ncov. Lancet. (2020) 395:311. 10.1016/s0140-6736(20)30186-0 31986259 PMC7134625

[10] Zheng G Jimba M Wakai S. Exploratory study on psychosocial impact of the severe acute respiratory syndrome (sars) outbreak on chinese students living in Japan. Asia Pac J Public Health. (2005) 17:124–9. 10.1177/101053950501700211 16425657

[11] Moldofsky H Patcai J. Chronic widespread musculoskeletal pain, fatigue, depression and disordered sleep in chronic post-sars syndrome; a case-controlled study. BMC Neurol. (2011) 11:37. 10.1186/1471-2377-11-37 21435231 PMC3071317

[12] Baglioni C Battagliese G Feige B Spiegelhalder K Nissen C Voderholzer U. Insomnia as a predictor of depression: a meta-analytic evaluation of longitudinal epidemiological studies. J Affect Disord. (2011) 135:10–9. 10.1016/j.jad.2011.01.011 21300408

[13] Frank S Gonzalez K Lee-Ang L Young MC Tamez M Mattei J. Diet and sleep physiology: public health and clinical implications. Front Neurol. (2017) 8:393. 10.3389/fneur.2017.00393 28848491 PMC5554513

[14] Tareq SR Likhon RA Rahman SN Akter S Basher MS Hasan MS. Depression among medical students of Bangladesh. Mymensingh Med J. (2020) 29:16–20. 31915330

[15] Koo DL Yang K I Kim J H Kim D Sunwoo J S Hwangbo Y. Association between morningness-eveningness, sleep duration, weekend catch-up sleep and depression among korean high-school students. J Sleep Res. (2020). 10.1111/jsr.13063. [Epub ahead of print]. 32391631

[16] Zhou HQ Yao M Chen G Y Ding X D Chen Y P Li DG. Functional gastrointestinal disorders among adolescents with poor sleep: a school-based study in Shanghai, China. Sleep Breath. (2012) 16:1211–8. 10.1007/s11325-011-0635-5 22203339

[17] Sahranavard S Esmaeili A Salehiniya H Behdani S. The effectiveness of group training of cognitive behavioral therapy-based stress management on anxiety, hardiness and self-efficacy in female medical students. J Educ Health Promot. (2019) 8:49. 10.4103/jehp.jehp_327_18 30993142 PMC6432834

[18] Lawrence J Ashford K Dent P Gender differences in coping strategies of undergraduate students and their impact on self-esteem and attainment. Active Learning in Higher Education. (2006) 7:273–81.

[19] Day AL Livingstone HA Gender differences in perceptions of stressors and utilization of social support among university students. Canad J Behav Sci. (2003) 35:73–83.

[20] Li CE DiGiuseppe R Froh J. The roles of sex, gender, and coping in adolescent depression. Adolescence. (2006) 41:409–15. 17225659

[21] Salfi F Lauriola M Amicucci G Corigliano D Viselli L Tempesta D. Gender-related time course of sleep disturbances and psychological symptoms during the Covid-19 lockdown: a longitudinal study on the italian population. Neurobiol Stress. (2020) 13:100259. 10.1016/j.ynstr.2020.100259 33102641 PMC7572275

[22] Taylor TS Raynard A L Lingard Perseverance L. Faith and stoicism: a qualitative study of medical student perspectives on managing fatigue. Med Educ. (2019) 53:1221–29. 10.1111/medu.13998 31657067

[23] Maheshwari G Shaukat F. Impact of poor sleep quality on the academic performance of medical students. Cureus. (2019) 11:e4357. 10.7759/cureus.4357 31192062 PMC6550515

[24] Shao R He P Ling B Tan L Xu L Hou Y. Prevalence of depression and anxiety and correlations between depression, anxiety, family functioning, social support and coping styles among chinese medical students. BMC Psychol. (2020) 8:38. 10.1186/s40359-020-00402-8 32321593 PMC7178943

[25] Romo-Nava F Tafoya S A Gutierrez-Soriano J Osorio Y Carriedo P Ocampo B. The association between chronotype and perceived academic stress to depression in medical students. Chronobiol Int. (2016) 33:1359–68. 10.1080/07420528.2016.1217230 27579890

[26] Michels N. Biological underpinnings from psychosocial stress towards appetite and obesity during youth: research implications towards metagenomics, epigenomics and metabolomics. Nutr Res Rev. (2019) 32:282–93. 10.1017/s0954422419000143 31298176

[27] Nakamura C Ishii A Matsuo T Ishida R Yamaguchi T Takada K. Neural effects of acute stress on appetite: a magnetoencephalography study. PLoS ONE. (2020) 15:e0228039. 10.1371/journal.pone.0228039 31968008 PMC6975544

[28] Sohrabi C Alsafi Z O'Neill N Khan M Kerwan A Al-Jabir A World health organization declares global emergency: a review of the 2019 novel coronavirus (Covid-19). Int J Surg. (2020) 10.1016/j.ijsu.2020.02.034 PMC7105032 32112977

[29] Kim D. Does paid vacation leave protect against depression among working americans? A national longitudinal fixed effects analysis. Scand J Work Environ Health. (2019) 45:22–32. 10.5271/sjweh.3751 30403822 PMC7610217

[30] Wahbeh H Nelson M. Irest meditation for older adults with depression symptoms: a pilot study. Int J Yoga Therap. (2019) 29:9–17. 10.17761/2019-00036 30354905

[31] Tran BX Vo LH Phan HT Pham HQ Vu GT Le HT Mobilizing medical students for covid-19 responses: experience of vietnam. J Glob Health. (2020) 10:020319 10.7189/jogh.10.020319 33110521 PMC7559422

